# A novel BRD4 inhibitor suppresses osteoclastogenesis and ovariectomized osteoporosis by blocking RANKL-mediated MAPK and NF-*κ*B pathways

**DOI:** 10.1038/s41419-021-03939-7

**Published:** 2021-06-26

**Authors:** Ying Liu, Wenjie Liu, Ziqiang Yu, Yan Zhang, Yinghua Li, Dantao Xie, Gang Xie, Li Fan, Shipeng He

**Affiliations:** 1 Institute of Translational Medicine, Shanghai University, 99 Shangda Road, Shanghai, 200444 China; 2 Department of Orthopedics, The Second Affiliated Hospital, Shantou University Medical College, 22 Xinling Road, Shantou, 515041 China; 3 School of Pharmacy, Naval Medical University, 325 Guohe Road, Shanghai, 200433 China

**Keywords:** Cell biology, Chemical biology, Osteoporosis

## Abstract

Bromodomain-containing protein 4 (BRD4) has emerged as a promising treatment target for bone-related disorders. (+)-JQ1, a thienotriazolodiazepine compound, has been shown to inhibit pro-osteoclastic activity in a BRD4-dependent approach and impede bone loss caused by ovariectomy (OVX) in vivo. However, clinical trials of (+)-JQ1 are limited because of its poor druggability. In this study, we synthesized a new (+)-JQ1 derivative differing in structure and chirality. One such derivative, (+)-ND, exhibited higher solubility and excellent inhibitory activity against BRD4 compared with its analogue (+)-JQ1. Interestingly, (-)-JQ1 and (-)-ND exhibited low anti-proliferative activity and had no significant inhibitory effect on RANKL-induced osteoclastogenesis as compared with (+)-JQ1 and (+)-ND, suggesting the importance of chirality in the biological activity of compounds. Among these compounds, (+)-ND displayed the most prominent inhibitory effect on RANKL-induced osteoclastogenesis. Moreover, (+)-ND could inhibit osteoclast-specific gene expression, F‐actin ring generation, and bone resorption in vitro and prevent bone loss in OVX mice. Collectively, these findings indicated that (+)-ND represses RANKL‐stimulated osteoclastogenesis and averts OVX-triggered osteoporosis by suppressing MAPK and NF-*κ*B signalling cascades, suggesting that it may be a prospective candidate for osteoporosis treatment.

## Introduction

Osteoporosis is a type of skeletal disorder characterised by damaged bone quality and elevated risk of fracture, reducing the life quality of older adults and aggravating social burdens [1]. Bone undergoes dynamic remodelling, which involves the processes of bone resorption and bone formation [2]. Abnormal osteoclast activity may disrupt bone remodelling, leading to osteopenic diseases [3, 4]. Therefore, administration of agents that suppress abnormal osteoclast activity could be an effective therapeutic strategy for osteoprotection.

Epigenetics involves heritable alterations in cellular phenotype or gene expression, which are not associated with underlying alterations of gene code. Epigenetic modulators, such as histone deacetylation, DNA modifications by methylation, and non-coding RNA, play a pivotal role in bone metabolic disorders [5]. BRD4 acts as a key epigenetic regulator that selectively recognises and binds to acetylated histones, thus converting the chromatin status into transcriptional elongation through RNA polymerase II (Pol II) [6]. Moreover, it is closely related to the regulation of gene transcription and mediates the expression of transcription genes associated with carcinomas [7, 8]. Dysfunction of BRD4 is correlated with development of bone*-*related diseases, and recent studies have reported the therapeutic efficacy of specific BRD4 inhibitors in bone-related diseases [9, 10].

(+)-JQ1 is a thienotriazolodiazepine compound that competitively binds to the acetyl-lysine-binding pocket in the bromodomain and extra-terminal domain (BET) proteins and suppresses the mobilisation of BET proteins to the acetylated histone tail of its target genes [11, 12]. It represents a typical class of BRD4 inhibitors and interferes with BRD4 function and is widely used to detect the biological function of BRD4. Previous reports have indicated that (+)-JQ1 displays potent anti-cancer efficacy both in vitro and in vivo [13, 14]. Recently, (+)-JQ1 was reported to inhibit osteoclast differentiation and abolish bone resorption by disrupting BRD4-dependent RANKL activation of the nuclear factor of activated T cell cytoplasmic 1 (NFATc1) transcription [15]. However, the poor druggability such as short half-life [16], restricted its entry into clinical trials. In our study, to improve the physicochemical properties of (+)-JQ1, a novel derivative (+)-ND was designed and synthesized. The results indicated that (+)-ND possesses higher solubility and inhibitory activities towards BRD4 than (+)-JQ1. Furthermore, we synthesized enantiomers (−)-JQ1 and (−)-ND to investigate the effect of chirality of compounds on the biological activity, which showed that these enantiomers exhibit low anti-proliferative activity and exert slight effect on RANKL-induced osteoclastogenesis compared with (+)-JQ1 and (+)-ND. Among these derivatives, (+)-ND displayed the most prominent inhibitory role in RANKL-induced osteoclastogenesis. We demonstrated that (+)-ND represses RANKL‐stimulated osteoclastogenesis in vitro and impedes bone loss in OVX mice. Furthermore, we elucidated the possible mechanisms and determined the effect of (+)-ND on MAPK and NF-*κ*B signalling pathways.

## Results

### Compound (+)-ND is a potent suppressor for both BRD4 and osteoclast formation

**Fig. 1:**
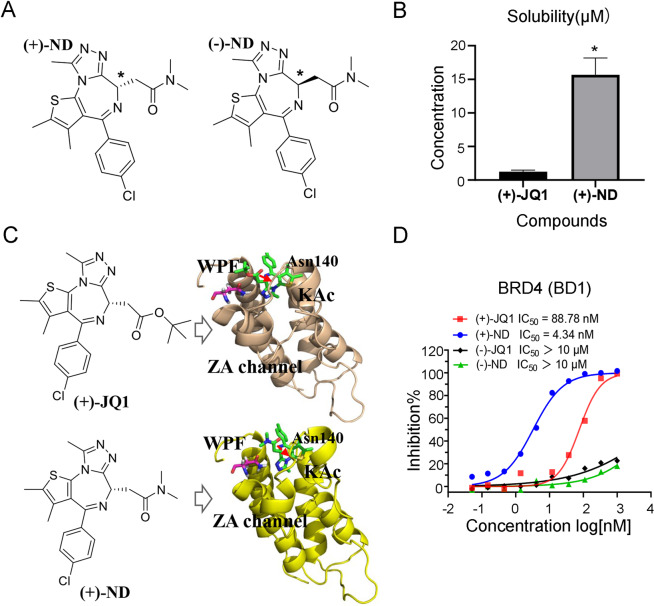
Identification of (+)-ND as a potent inhibitor of BRD4. **A** The structures of enantiomers (+)-ND and (-)-ND. **B** The water solubility of (+)-JQ1 and (+)-ND. ^*^P < 0.05 relative to the control. **C** The binding models of (+)-JQ1 and (+)-ND with BRD4 (PBD: 3MXF), respectively. **D** The inhibitory activities of different enantiomers toward BRD4 (BD1).

**Fig. 2:**
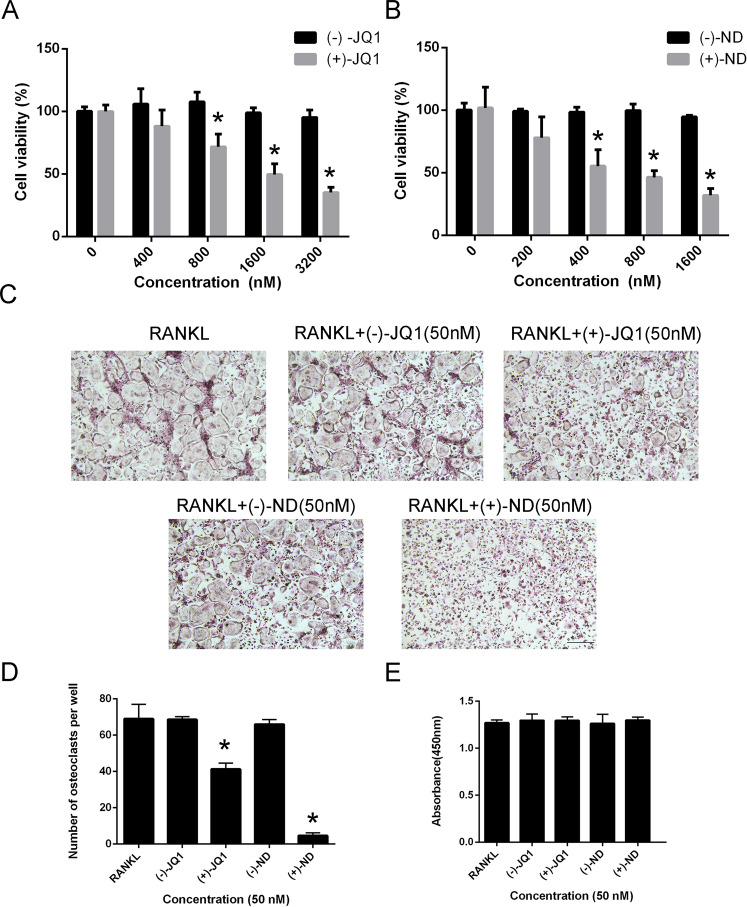
Compounds suppresses RANKL-induced osteoclast formation with no cytotoxicity in vitro. Effects of (+)-JQ1, (-)-JQ1 (**A**), (+)-ND, (-)-ND (**B**) on viability of BMMs at 72 h (*n* = 3). **C** BMMs were stimulated with RANKL in the presence of (+)-JQ1, (-)-JQ1, (+)-ND, or (-)-ND (50 nM) and stained for TRAP detection. **D** Quantification analysis of TRAP-positive multinucleated (nuclei ≥ 3) cells. **E** Cytotoxic effects of (+)-JQ1, (-)-JQ1, (+)-ND, or (-)-ND (50 nM) on BMMs were evaluated by CCK-8 analysis at 96 h. Error bars are indicated as mean ± SD. ^*^P < 0.05 compared with controls.

Improvement of pharmacological activities is related to physicochemical properties and membrane permeability of compounds [17, 18]. To improve the water solubility of (+)-JQ1, compound (+)-ND was then synthesized by replacing tertiary butyl with dimethyl amine (Fig. 1A and Supplementary Fig. 1). As a result, the solubility of (+)-ND was increased nearly 15-fold than that of (+)-JQ1 (Fig. 1B). The HTRF assays showed that introducing of dimethyl amine leading to better BRD4 (BD1) inhibitory efficacy with ~20-fold improvement of in vitro potency over (+)-JQ1 (Fig. 1D). In addition, the binding of (+)-ND with BRD4 was investigated through molecular docking to explain the inhibitory activities of enzymes. As shown in Fig. 1C and Supplementary Fig. 2, (+)-ND binds to the same pocket in BRD4 protein and displays a binding mode extremely similar to that of (+)-JQ1. The triazole ring of (+)-ND fitted well into the KAc docking pocket and formed hydrogen bonding interactions with conserved residue Asn140. The thiophene and phenyl group occupied the ZA channel and WPF regions, stabilized by hydrophobic interactions, respectively. Previous studies revealed that enantiomers of chiral compound exhibited great differences in inhibitory activities of target protein and cell proliferation [19]. Therefore, the enantiomers, (−)-JQ1 and (−)-ND, were synthesized to further assess their biological activity. As shown in Fig. 1D, the inhibitory activity of compounds (−)-JQ1 and (−)-ND against BRD4 (BD1) was significantly lower than that of their respective enantiomers (+)-JQ1 and (+)-ND. In addition, compared with their enantiomers, either (+)-JQ1 or (+)-ND markedly repressed the proliferative ability of BMMs in a dose‐dependent manner (Fig. 2A and B). Furthermore, BMMs were grown with 50 nM of (+)-JQ1, (−)-JQ1, (+)-ND, and (−)-ND to synthesise osteoclasts in the presence of M*-*CSF and RANKL. We observed that (+)-ND strongly repressed RANKL-induced osteoclastogenesis without cytotoxicity (Fig. 2C-E).

### (+)-ND suppresses RANKL-induced osteoclastogenesis in vitro

**Fig. 3:**
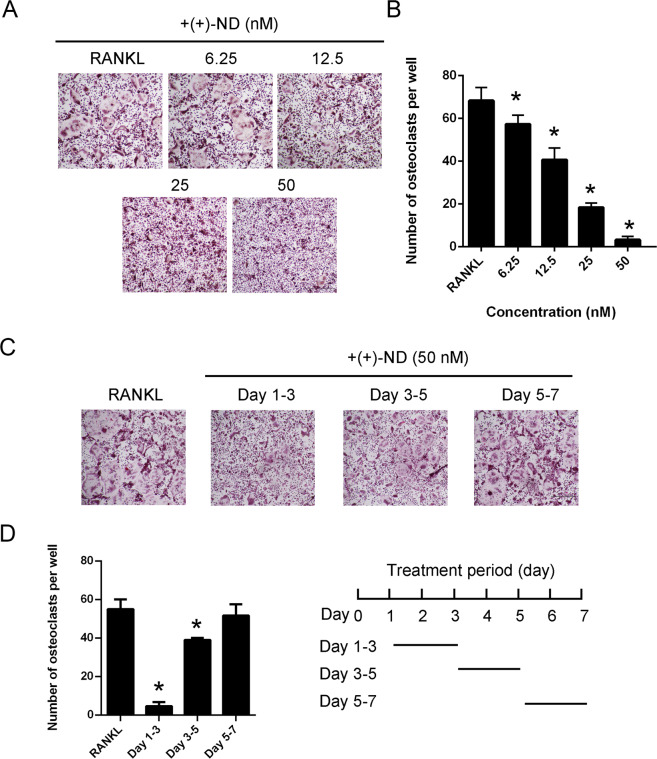
(+)-ND represses RANKL-induced osteoclast differentiation in vitro. **A** BMMs were stimulated with 50 ng/mL RANKL without or with indicated concentrations of (+)-ND (0, 6.25, 12.5, 25, or 50 nM) for 7 d. Osteoclasts were stained with TRAP. Scale bar = 100 μm. **B** The count of TRAP-positive multinucleated cells with ≥ 3 nuclei was quantified. ^*^P < 0.05 compared with controls. **C** BMMs were cultured with 50 nM (+)-ND on day 1–3, 3–5, or 5–7 in the presence of M-CSF (30 ng/mL) and RANKL (50 ng/mL). Osteoclasts were assessed via TRAP staining. **D** TRAP-positive multinucleated cell numbers with three or more nuclei and the area of osteoclasts per well were quantified. The findings presented represent at least 3 independent experiments. ^*^P < 0.05 in contrast with the control.

The effect of (+)-ND was assessed during RANKL-induced osteoclast formation. As shown in Fig. 3A and B, (+)-ND dose-dependently repressed TRAP-positive multinucleated osteoclast formation. Besides, to establish the inhibitory influence of (+)-ND on the process of osteoclastogenesis, we treated BMMs with 50 nM of (+)-ND at the early (1–3 days), intermediate (3–5 days), and late (5–7 days) stages of RANKL stimulation. (+)-ND strongly repressed the number and size of osteoclasts in its early stage but displayed trivial effect in the late stages of the process (Fig. 3C and D). Collectively, these data showed that (+)-ND represses osteoclast formation without cytotoxic effects.

### (+)-ND impairs the generation of F-actin ring and osteoclastic bone resorption in vitro

**Fig. 4:**
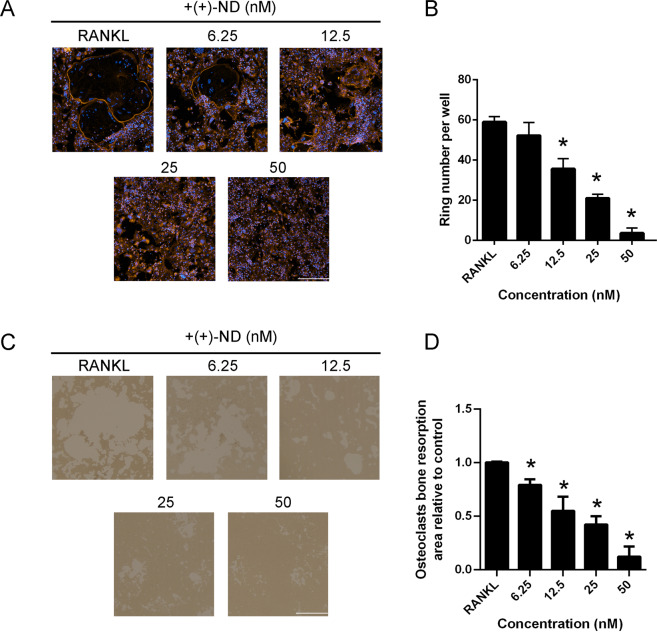
(+)-ND represses F-actin ring formation and osteoclastic resorption ability. **A** Osteoclasts were cultured with M-CSF (30 ng/mL), RANKL (50 ng/mL) and different concentrations of (+)-ND (0, 6.25, 12.5, 25 or 50 nM) for 7 d, stained with TRITC phalloidin and then assessed by immunofluorescence microscopy. DAPI was employed to stain the nuclei. Scale bar, 100 μm. **B** The number of actin ring at each (+)-ND concentration was counted. *n* = 3; ^*^P < 0.05 compared with the controls. **C** Representative images of mature osteoclasts grown in hydroxyapatite-coated plates incubated with RANKL, as well as M-CSF with or without (+)-ND. Scale bar, 100 μm. **D** Image J software was employed to quantify the resorption pit areas. ^*^P < 0.05 relative to the control.

Generation of F-actin ring is the most visible feature of mature osteoclasts in osteoclastogenesis. We observed that BMMs could form typical F-actin ring structures following RANKL stimulation in the control group. Consistent with previous TRAP staining results, treatment with (+)-ND significantly decreased the size and number of F-actin ring structures in a dose-dependent manner, implying that (+)-ND repressed the F-actin ring generation by mature osteoclasts (Fig. 4A and B).

The osteoclast actin ring generation in RANKL-induced osteoclasts is required for osteoclast bone resorption. Therefore, we evaluated whether (+)-ND treatment affected bone resorptive function of mature osteoclast. In comparison with controls, exposure of cells to (+)-ND in a dose dependent approach diminished bone resorbed areas of mature osteoclasts (Fig. 4C and D), suggesting that (+)-ND repressed bone resorption function of osteoclasts in vitro.

### (+)-ND attenuates the osteoclast marker genes expression during osteoclastogenesis

**Fig. 5:**
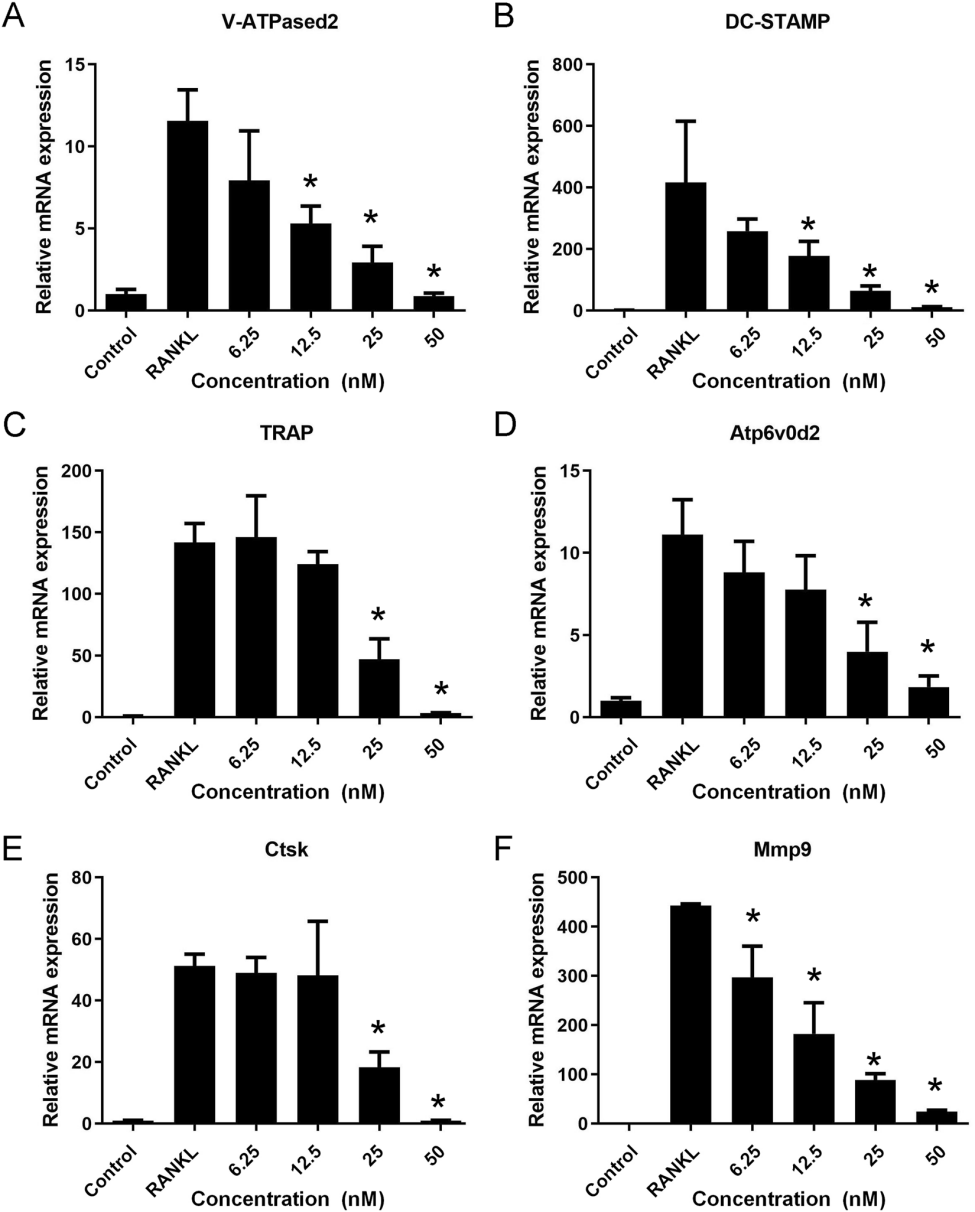
(+)-ND inhibits osteoclast marker gene expression. **A–F** BMMs were cultured with M-CSF (30 ng/mL) and RANKL (50 ng/mL) with indicated concentrations of (+)-ND. Osteoclast-distinct gene expression (for V-ATPase d2 (**A**), DC-STAMP (**B**), TRAP (**C**), Atp6v0d2 (**D**), Ctsk (**E**), and Mmp9 (**F**)) was examined by qRT-PCR. The expression level of correlated genes was normalized to GAPDH. ^*^P < 0.05 in contrast with the control group.

To elucidate the function of (+)-ND on osteoclast differentiation, osteoclastic genes expression was explored through quantitative PCR. Results revealed that osteoclast biosignature genes, consisting of V-ATPase d2, DC-STAMP, TRAP, Atp6v0d2, Ctsk, and Mmp9 were up-regulated in the RANKL-induced control group, whereas (+)-ND repressed the expression of the same marker genes during the process of osteoclastogenesis (Fig. 5A-F). Therefore, these data demonstrated that (+)-ND suppresses osteoclastogenesis by repressing RANKL-induced osteoclast-distinct gene expression.

### (+)-ND represses RANKL-induced activation of MAPK and NF-*κ*B signalling pathways

**Fig. 6:**
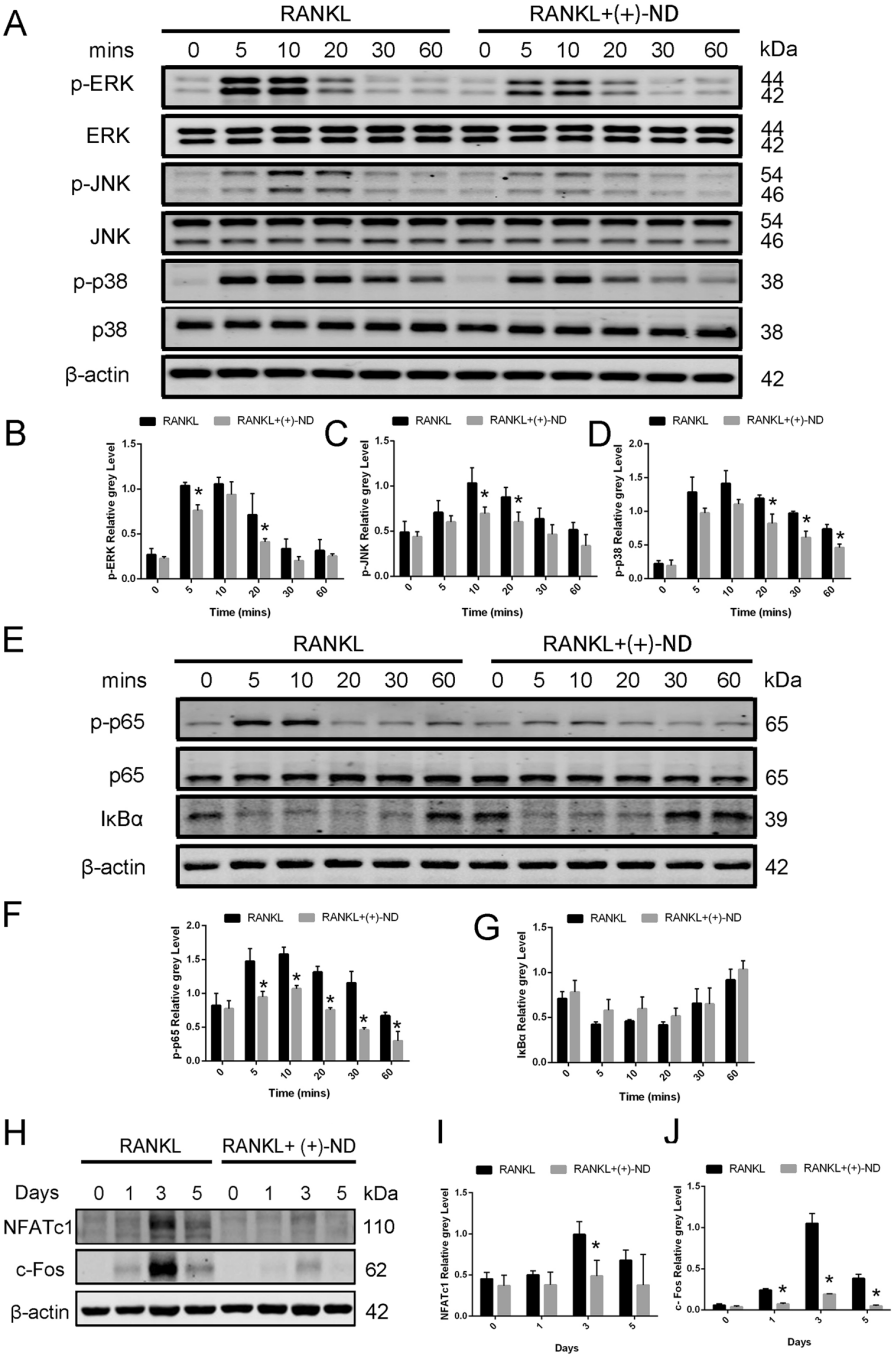
(+)-ND represses the activation of MARK and NF-*κ*B pathways by RANKL stimulation. **A** We pretreated BMMS with or without (+)-ND (50 nM) in serum-free medium for 2 h. Thereafter, RANKL (50 ng/mL) was employed to stimulate cells. Western Blotting was carried out with specific antibodies against p-ERK, ERK, p-JNK, JNK, p-p38, p38 to analyze cell lysates. Total β-actin was employed as the loading control. Representation of the ratio of p-ERK versus ERK (**B**), p-JNK versus JNK (**C**), p-p38 versus p38 (**D**) were shown. Band intensity was determined by Image J. **E** The activity of NF-*κ*B signal pathway was evaluated by Western Blotting and β-actin was employed as the loading control. The ratio of p-p65 versus p65 (**F**), and I*κ*Bα versus β-actin (**G**) were quantified using Image J. **H** BMMs were exposed to (+)-ND (50 nM) for 0, 1, 3, and 5 d. Cell lysates were explored via Western Blotting with distinct antibodies against NFATc1 and c-Fos. Total β-actin was employed as the loading control. The protein expression contents of NFATc1 (**I**) and c-Fos (**J**) were quantified by Image J. The data were confirmed at least three times (^*^P < 0.05 relative to the control group at indicated times).

To assess the underlying mechanisms by which (+)-ND suppressed osteoclast differentiation in vitro, we examined early MAPK signalling cascade activation, as well as NF-*κ*B pathway mediated by RANKL. As shown in Fig. 6A–D, the early MAPK activation was demonstrated through ERK, JNK, and p38 phosphorylations in response to RANKL, which was remarkably attenuated following after exposure to (+)-ND. Regarding early NF-*κ*B signalling activation, RANKL-induced I*κ*Bα degradation, and the p65 phosphorylation were remarkably repressed by (+)-ND treatment (Fig. 6E–G).

MAPK and NF-*κ*B activations participate in the subsequent induction of NFATc1 and c-Fos. These transcription factors are needed for differentiation and maturation of osteoclasts [20, 21]. Herein, we established the expression of protein contents of NFATc1 and c-Fos peaks at day 3 after RANKL stimulation. On the contrary, (+)-ND treatment remarkably repressed the expression of these proteins for the same period (Fig. 6H-J). Consequently, our data implied that (+)-ND suppresses differentiation of osteoclasts through repressing MAPK and NF-*κ*B signalling pathways.

### (+)-ND attenuates OVX-induced bone loss in vivo

**Fig. 7:**
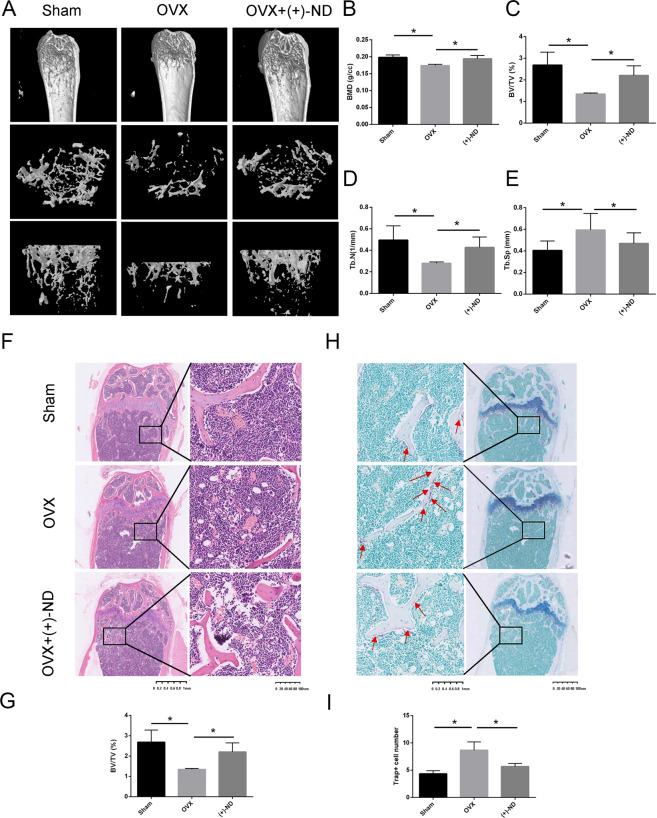
(+)-ND prevents OVX-induced bone loss in vivo. **A** Representative micro-CT photographs of the distal femur form sham-treated controls (sham), OVX with PBS injection (OVX), OVX with 10 mg/kg (+)-ND. Quantitative assessment of BMD (**B**), BV/TV (%) (**C**), Tb. N (**D**), and Tb.Sp (**E**) in each group. **F** Representative photographs of H&E staining from each treatment group. **G** Data of the BV/TV were shown in the bar chart. (*n* = 3). **H** Representative images of TRAP staining for the three groups were shown. **I** The number of TRAP‐positive multinucleated osteoclasts on the trabecular bone surface were shown in the bar chart. All bar graphs present the mean ± SD; ^*^P < 0.05 when relative to OVX group.

Considering the inhibitory effect of (+)-ND on osteoclastogenesis in vitro, we investigated the therapeutic significance of (+)-ND in the OVX-induced bone loss murine model. The result of micro-computed tomography (micro-CT) revealed that compared with the OVX group, (+)-ND reduced bone mass loss in the distal femur (Fig.7A). Quantitative analysis showed that injections of (+)-ND (10 mg/kg) in OVX mice remarkably increased the values of BMD, BV/TV, as well as Tb.N and diminished the values of Tb.Sp (Fig. 7B–E). The histological assessment further verified that OVX-induced bone loss was abolished when exposed to (+)-ND (10 mg/kg) every other day (Fig. 7F and G). Moreover, the findings of TRAP staining were consistent with the data of micro-CT and indicated that (+)-ND remarkably diminishes TRAP-positive cell count around the trabecula compared with OVX mice (Fig. 7H and I). Collectively, our findings revealed that (+)-ND represses osteoclast formation and impedes OVX-induced bone loss.

## Discussion

Bone homoeostasis is a dynamic balance that is maintained primarily by bone generation and resorption. Over-activated osteoclasts disrupt the bone microarchitecture, which results in bone loss-linked diseases, such as osteoporosis and rheumatoid arthritis [22, 23]. Currently, available agents used for treating osteoporosis in the clinic exhibit side effects after long-term administration. For example, bisphosphonates suppress bone formation and result in nonunion after fracture [24, 25]. Further investigation has shown that (+)-JQ1 repressed osteoclast differentiation and pathological bone loss in a post-menopausal osteoporosis model [26, 27]. However, the clinical application of (+)-JQ1 could be limited given its solubility and short half-life [28]. In order to explore potential therapeutics for treating osteoporosis in substitute of current available candidates, we designed and synthesized (+)-ND, which is a (+)-JQ1 derivative, by utilizing structure-based drug design strategy.

Epigenetics refers to functionally relevant modification to the genome that associates with histone modifications, DNA methylation, as well as non-coding RNA, and have been demonstrated taking critical roles in bone regeneration [29]. BET proteins (BRD2, BRD3, BRD4, and the testis-specific BRDT) serve as epigenetic reader that mainly recognize acetylated lysine on histone tails to regulate chromatin accessibility [30]. Specifically, BRD4 has been recognized as a key regulator during cancer progression owing to its role in gene expression regulation [31], making it a popular cancer treatment target. Indeed, the first reported BRD4 suppressor (+)-JQ1, has been shown to be effective in many preclinical human disease models [32, 33]. Investigation has shown that (+)-JQ1 repressed osteoclast differentiation and pathological bone loss in a post-menopausal osteoporosis model. In our study, we further improved the applicability of BRD inhibitor by designing and synthesizing (+)-ND, the derivative (+)-JQ1. Our results not only revealed that (+)-ND exhibited better solubility and stronger inhibition effect on RANKL-induced osteoclastogenesis without cytotoxicity comparing to (+)-JQ1, but also presented the incapabilities of (-)-JQ1 and (-)-ND in anti-proliferative function and suppressive effects on RANKL-induced osteoclastogenesis, emphasizing the chirality-specific efficacy of (+)-ND. Moreover, in vitro assays confirmed the direct repressive influence of (+)-ND on RANKL-stimulated osteoclast generation and osteoclast bone resorption derived from mature BMMs.

Osteoclast differentiation and formation is dependent on activation via two key cytokines, M-CSF and RANKL [34, 35]. Binding of RANKL to RANK induced recruitment of TRAF6, which stimulates downstream MAPK and NF-*κ*B signal pathways required for osteoclastogenesis [36]. In this study, (+)-ND treatment remarkably attenuated RANKL-stimulated phosphorylation of ERK and JNK as well as repressed degradation of I*κ*Bα and p65 phosphorylation, indicating that (+)-ND suppressed osteoclastogenesis partially attributed to the repression of MAPK and NF-*κ*B signalling pathways. Previous studies demonstrated that c‐Fos and NFATc1 are two critical transcriptional factors, which may bind to osteoclast-specific gene promoters, constituting TRAP, calcitonin receptor (CTR), and Ctsk, and lead to the development of multinucleated bone-resorbing osteoclasts ultimately [37]. We observed that (+)-ND repressed the expression levels of c-Fos and NFATc1, consequently suppressed the expression of osteoclast-distinct genes, such as V-ATPase d2, TRAP, DC-STAMP, Atp6v0d2, Ctsk, and Mmp-9. All of these results suggested that (+)-ND influences the MAPK and NF-*κ*B signalling pathways, leading to the suppression of NFATc1 activation, and therefore, of osteoclastogenesis. However, these data only explored the molecular changes in in vitro setting, which could not mimic the adaptable characteristics of natural bone environment, making in vivo experiments a potential direction of future research.

Based on these in vitro findings, we additionally explored whether (+)-ND has promising therapeutic influence on OVX mice model. It has been reported to protect mice against OVX-stimulated bone loss in vivo though repressing osteoclast generation, as well as bone resorption. The assessment of micro-CT scanning and H&E staining on the femur showed that OVX-induced bone loss was remarkably diminished after (+)-ND treatment. Besides, TRAP staining demonstrated that positive cells were remarkably reduced by (+)-ND treatment. These data collectively suggested that (+)-ND exhibits a considerable protective influence on OVX-induced bone loss. However, another major limitation of this study is the lack of clinical data on the safety and efficacy of this potential drug which indicates the needs of further investigations to evaluate the efficacy of this novel agents in treating osteoporosis in human.

In summary, our study reveals that (+)-ND can repress osteoclast synthesis, bone resorption function in vitro, and attenuate OVX-triggered bone loss in vivo. The anti-osteoclastogenic effect of (+)-ND is elicited primarily through inhibiting RANKL-mediated MAPK and NF-*κ*B activation, together with down-regulating NFATc1 and c-Fos expressions. Here, we demonstrate that (+)-ND could be a prospective therapeutic candidate against osteoporosis.

## Materials and methods

### Chemistry

Unless otherwise noted, all reagents were obtained from commercial suppliers and used without further purification. All starting materials were commercially available. ^1^H-NMR spectra were documented on AVANCE300 or AVANCE600 Bruker spectrometer (Leipzig, Bruker Company, Germany), and chemical shifts were documented in ppm downfield from tetrame-thylsilane (TMS). The coupling constants (*J*) were documented in Hz. Moreover, the mass spectra (MS) were determined on a Waters Acquity UPLC. TLC assessment was conducted on silica gel plates GF254 (Qingdao Haiyang Chemical, China). Besides, silica gel column chromatography was accomplished using Silica gel 60 G (Qingdao Haiyang Chemical, China). The purities of assayed compounds were greater than 95%, as estimated by reversephase (RP) HPLC analysis.

### Biological experiment

R&D (Minneapolis, MN, United States) provided the recombinant murine macrophage colony-stimulating factor (M-CSF), as well as the recombinant murine receptor activator of NF-*κ*B ligand (RANKL), whereas α-minimum essential medium (*α*-MEM) along with foetal bovine serum (FBS) were provided by Hyclone (Logan, UT, US). Cell Counting Kit-8 (CCK-8) was obtained from Beyotime (Shanghai, China). TRAP staining kit was bought from Sigma-Aldrich (St. Louis, MO, United States). Anti-extracellular signal-related kinase (ERK) (Cat#: 4695, 1:1000), anti-I*κ*Bα (Cat#: 4812, 1:1000), anti-p-JNK (Cat#: 4668, 1:1000), anti-c-Jun N-terminal kinase (JNK) (Cat#: 9252, 1:1000), anti-p65 (Cat#: 8242, 1:1000), anti-p-ERK (Cat#: 4370, 1:2000), anti-p-p38 (Cat#: 4511, 1:1000), anti-c-Fos (Cat#: 2250, 1:1000), anti-p-p65 (Cat#: 3033, 1:2000), anti-p38 (Cat#: 8690, 1:1000), and anti-β-actin (Cat#: 8457, 1:1000), together with the fluorescence-labelled secondary antibodies against rabbit (Cat#: 5151, 1:30000) or mouse IgG (Cat#: 5470, 1:15000) were provided by Cell Signalling Technology (Danvers, MA, USA). TRITC phalloidin was obtained from Solarbio Life Sciences (Beijing, China). Prime Script RT Master Mix and TB Green Premix Ex Taq were from Takara Bio Inc. (Shiga Prefecture, Japan).

### BMMs isolation and culture

Primary bone marrow monocytes (BMMs) were obtained from the bone marrow of 5-week-old, female C57BL/6 mice. Briefly, the mice were sacrificed, and cells were flushed from the femoral and tibial bones. After centrifuge, cells were cultured in *α*-MEM medium comprising 10% FBS and 1% penicillin/streptomycin (complete *α*-MEM) supplemented with 30 ng/mL M-CSF. After 4 d of culture, the cells were washed gently by PBS, and the adherent cells were harvested as BMMs for further experiments. All cells were incubated in a 37 °C incubator with 5% CO_2_.

### Cell viability assay

BMMs (8 × 10^3^ cells/well) were seeded in 96-well plates overnight. All cells were subsequently replaced with fresh culture medium and treated with increasing concentrations of (+)-ND (0, 6.25, 12.5, 25 or 50 nM) for 96 h. A 10 μL volume of CCK-8 reagent was added to each well, and the plate was incubated for 2 h in the dark under 37 °C. Absorbance was measured at 450 nm using the Cytation five Cell Imaging Multi-Mode Reader (BioTek, Vermont, United States).

### Osteoclast differentiation in vitro

To generate osteoclast, BMMs were seeded in 96-well plates at a density of 8 × 10^3^ cells per well in *α*-MEM containing 30 ng/mL M-CSF and 50 ng/mL RANKL without or with indicated concentrations of (+)-ND (0, 6.25, 12.5, 25 or 50 nM). Cell media was replaced every 2 d. After 7 d of culture, cells were washed twice with PBS and fixed with 4% paraformaldehyde (PFA) for 15 min, then stained with TRAP solution at 37 °C for 30 min. Each well was assessed using a microscope, and the number of TRAP-positive cells was counted. The average size of mature osteoclasts was quantified using ImageJ software (National Institutes of Health, Bethesda, MD).

### F-actin ring fluorescent staining

M‐CSF‐dependent BMMs were seeded in 96-well plates at a density of 8 × 10^3^ cells/well in complete *α*-MEM supplemented with (+)-ND (0, 6.25, 12.5, 25 or 50 nM), 30 ng/mL of M-CSF, and 50 ng/mL of RANKL. Cell media was replaced every other day for 7 d. Cells were then fixed with 4% PFA for 15 min, permeated with 0.1% (v/v) Triton X-100 in PBS for 15 min and enclosed with 0.25% bovine serum albumin (BSA) at room temperature for 1 h. After washing, cells were incubated with TRITC phalloidin for 2 h at room temperature and then counterstained with DAPI for 10 min in the dark. Following washing with PBS twice, fluorescence images were captured using fluorescence microscopy.

### Bone resorption assay

M-CSF dependent BMMs were incubated in 6-well culture plates stimulated with RANKL. Once small preosteoclast-like cells formed, cells were digested and re-planted on hydroxyapatite-coated plates (8 × 10^3^ cells/well) with 30 ng/mL of M-CSF and 50 ng/mL of RANKL. After 8 h, the adherent cells were exposed to (+)-ND (0, 6.25, 12.5, 25 or 50 nM). After 4 d, the adherent cells exposed to 5% sodium hypochlorite solution for 15 min, rinsed twice with sterile water, air-dried for 4 h. We imaged the osteoclast-resorbing area by the Nikon microscope (Nikon Corporation, Minato, Tokyo, Japan) and the Image J software employed to quantify the area of bone resorption. The experiments were performed independently thrice.

### qRT-PCR

BMMs were seeded in 6-well plates at a density of 2 × 10^5^ cells /well and cultured with 30 ng/mL M-CSF, and 50 ng/mL RANKL in the absence or presence of (+)-ND (0, 6.25, 12.5, 25 or 50 nM). Once the osteoclasts generated in control group, total RNA was isolated using TRIzol reagent (Thermo Fisher Scientific). 1 μg of RNA was reverse-transcribed into cDNA using PrimeScript RT reagent Kit. Then, 100 ng of cDNA, 10 μL of TB Green Premix Ex Taq, and 10 pmol of specific primers in 20 µL of RT‐PCR reaction buffer were used for amplification. The reaction conditions were as follows: 5 min under 95 °C; 10 s under 95 °C, 20 s under 60 °C, and 20 s under 72 °C for 30 cycles; and 10 min of elongation under 72 °C. Glyceraldehyde-3-phosphate dehydrogenase (GAPDH) was used as an internal control. Data were normalized to GAPDH using 2^-ΔΔCT^ method. The primers were listed in Supplementary Materials and Methods.

### Protein extraction and western blotting

Freshly isolated BMMs were seeded in 6-well plates (5×10^5^cells/well) and allowed to adhere overnight. Next day, cells were serum starved for 2 h, preconditioned with or without 50 nM (+)-ND for 2 h. Then the cells were stimulated with RANKL for 0 min, 5 min, 10 min, 20 min, 30 min, and 60 min. Cells were rinsed three times in PBS, protein was extracted using RIPA buffer containing protease and phosphatase inhibitors. Protein content was measured with BCA protein assay kit. Equal amounts of protein (30 µg) were electrophoresed on 10% SDS-PAGE gels and transferred to nitrocellulose membranes. The membranes were blocked with 5% non-fat dry milk in TBST (pH 7.5, 0.15 M NaCl, 0.05 M Tris–HCl, and 0.2% Tween-20) for 2 h at room temperature. The membranes were washed in TBST and incubated with primary antibodies at 4 °C overnight. The next day after TBST rinsing, corresponding secondary antibodies were then incubated for 1 h. Bands were observed through the ChemiDoc^TM^ MP imaging system (Bio-Rad, Hercules, CA, USA). Quantitative analysis of bands’intensity using Image J software.

### OVX-induced osteoporosis mouse model

Twenty-four 8-weeks old female C57BL/6 mice were provided by the CAVENS Laboratory Animal (Changzhou, china). The mice were grouped randomly into 3 groups (*n* = 6/group): the sham that received PBS injection (Sham group), OVX that received PBS injection (OVX group), OVX with 10 mg/kg (+)-ND injection. According to standard procedures, the mice in OVX + ( + )-ND group and OVX group were intraperitoneally injected with 3% chloral hydrate under anaesthesia and underwent bilateral ovariectomy to remove ovaries. The mice in sham group received surgery but ovaries were not removed. One week for postoperative recovery after surgery, we subcutaneously injected the mice with PBS (Sham and OVX groups) or with (+)-ND (10 mg/kg) every 2 d. After 6 weeks, we euthanized all the mice, and all the femurs were collected, fixed by 4% PFA, and prepared for the micro-CT and histologic evaluations.

### Microcomputed tomography analyses

A random side femur from each mouse was scanned by microcomputed tomography (micro-CT, bruker skyscan1176, Germany). The following scanning parameters were set: source current, 450 μA; source voltage, 50 kV; rotation step, 0.40 degree; pixel size, 8.96 µm; and AI, 0.5 mm filter. A region of interest (ROI) 0.5 mm over the dismal femur growth plate. Several bone-related parameters were analyzed, including the bone mineral density (BMD), trabecular number (Tb.N), bone volume/total volume (BV/TV), as well as trabecular separation (Tb.Sp, mm).

### Histomorphometric examination

For the histological analysis of the bones, 4% PFA was employed to fix the femurs for 2 d, and then 10% EDTA (pH = 7.4) solution used to decalcify them for 2 weeks. Following decalcification, samples were collected and embedded in paraffin, followed by sectioning through a microtome (5-μm-thick sections). We stained these sections using hematoxylin, as well as eosin (H&E) or TRAP. The Nikon microscope (Nikon Corporation, Minato, Tokyo, Japan) was used to capture images.

### Statistical analyses

All data are indicated as mean ± SD of at least three different assays. Statistical were carried out by the two-tailed Student’s *t* test or the one-way analysis of variance. *P* < 0.05 signified statistical significance.

## Supplementary information

Supplementary Materials and Methods

Supplementary figure legends

Figure S1

Figure S2
