# Supplementary material for: A novel BRD4 inhibitor suppresses osteoclastogenesis and ovariectomized osteoporosis by blocking RANKL-mediated MAPK and NF-κB pathways
Source: Cell Death Dis. 2021 Jun 26;12(7):654. doi: 10.1038/s41419-021-03939-7 (PMC8236062; doi:10.1038/s41419-021-03939-7)

**Supplemental information**

**A novel BRD4 inhibitor suppresses osteoclastogenesis and ovariectomized osteoporosis by blocking RANKL-mediated MAPK and NF-*κ*B pathways**

**Table of Contents**

[Experimental protocols of biological assays S3](#_Toc24892712)

[The primers of qRT-PCR S7](#_Toc24892712)

[Reference S9](#_Toc24892713)

[Spectral data S10](#_Toc24892714)

**Experimental protocols of biological assays**

**TR-FRET Bromodomain Binding Assay.** This assay was conducted by Jinan Huawei pharmaceutical Co. Ltd., China. The assay was performed by TR-FRET technology using a recombinant BRD4 (BD1) subtype and its corresponding ligand. The TR-FRET signal from the assay is correlated with the amount of ligand binding to the bromodomain. The compounds final concentration of DMSO is 1 % in all reactions. All of the binding reactions were conducted at room temperature. The 20 µL reaction mixture in assay buffer contains BRD, BET ligand and the indicated amount of inhibitor. For the negative control, 5 μL of the assay buffer was added instead of the BET ligand. The reaction mixture incubated for 120 min. After the incubation with the ligand, TR-FRET signal was measured using Tecan Infinite M1000 plate reader.

**Molecular docking.** The crystal structure of BRD4 was obtained from protein database bank (PDB ID: 3MXF^1^) and prepared for docking using the protein preparation tool in Discovery Studio 3.0. During this process, the ligands and waters were removed and hydrogens were added to the structure. Staged minimization was performed with default setting. The docking studies were carried out using GOLD 5.0. Binding site was defined as whole residues within a 10 Å radius subset encompassing the ligand. Conformations were generated by genetic algorithm and scored using GoldScore as fitness function. The best conformation was chosen to analyze the ligand–protein interaction.

**Synthetic Procedures**

**(2-Amino-4,5-dimethylthiophen-3-yl)(4-chlorophenyl) methanone (2)**

To a solution of 4-chlorobenzoyl acetonitrile (2.48 g, 13.80 mmol), 2-butanone (1.34 mL, 13.80 mmol) and morpholine (1.20 mL, 13.80 mmol) in ethanol (20 mL) was added Sulfur (0.44 g, 13.80 mmol). The solution was stirred at 70 ºC for 12 h. After cooling to room temperature, the solvent was evaporated and the residue was poured into water (50 mL). The aqueous layer was extracted with EtOAc (20 mL × 3). The combined organic layer was washed with brine (20 mL), dried over anhydrous Na_2_SO_4_, and concentrated under the reduced pressure. The residue was purified by column chromatography on silica gel (Hexane: EtOAc = 20: 1) to give compound **2** as a yellow solid (4.53 g, 91.3%). ^1^H NMR (DMSO-*d*_6_, 300 MHz) *δ*: 1.45 (s, 3H), 2.06 (s, 3H), 7.42 (d, *J =* 8.43 Hz, 2H), 7.50 (t, *J =* 8.43 Hz, 2H), 7.94 (s, 2H).

**(*S*)-*tert*-Butyl-3-((((9H-fluoren-9-yl)methoxy)carbonyl)amino)-4-((3-(4-chloro benzoyl)-4,5-dimethylthiophen-2-yl)amino)-4-oxobutanoate (3)**

To the solution of Fmoc-Asp-OtBu (8.32 g, 20 mmol), HBTU (7.59 g, 20 mmol) and DIPEA (7.2 mL, 40 mmol) in DMF (5 mL) was added **2** (2.66 g, 10 mmol). The resulting mixture was stirred at room temperature for 24 h, and then water (50 mL) was added. The aqueous layer was extracted with EtOAc (20 mL × 3). The combined organic layer was washed with brine (20 mL), dried over anhydrous Na_2_SO_4_, and concentrated to a tan oil. Purification by column chromatography on silica gel (Hexane: EtOAc = 10: 1) gave compound **3** as a yellow solid (4.95 g, 75%). ^1^H NMR (DMSO-*d*_6_, 300 MHz) *δ*: 1.35 (s, 9H), 1.38 (d, *J =* 10.72 Hz, 2H), 1.74 (s, 3H), 2.25 (s, 3H), 2.47 (d, *J =* 8.71 Hz, 1H), 4.21 (d, *J =* 6.62 Hz, 1H), 4.25-4.34 (m, 1H), 7.46 (dd, *J =* 13.51 Hz, 8.01 Hz, 1H), 7.25-7.34 (m, 2H), 7.40 (t, *J =* 7.98 Hz, 2H), 7.51 (d, *J =* 8.27 Hz, 2H), 7.57 (d, *J =* 8.27 Hz, 2H), 7.68 (d, *J =* 4.96 Hz, 2H), 7.88 (d, *J =* 7.45 Hz, 2H), 7.94 (d, *J =* 8.27 Hz, 1H), 10.99 (s, 1H).

**(*S*)-*tert*-Butyl-2-(5-(4-chlorophenyl)-6,7-dimethyl-2-oxo-2,3-dihydro-1*H*-thieno**

**[2,3-*e*][1,4]diazepin-3-yl) acetate (4)**

To a solution of **3** (4.27 g, 6.48 mmol) in DMF (10 mL) was added piperidine (5 mL). The reaction solution was stirred at room temperature for 1 h, and then water (50 mL) was added to the mixture. The aqueous layer was extracted with EtOAc (20 mL × 3). The combined organic layer was washed with brine (20 mL), dried over anhydrous Na_2_SO_4_, and evaporated under reduced pressure to give a pale yellow solid, which was used for the next step without further purification. The crude product (3.12 g, 7.22 mmol) was dissolved in toluene (10 mL). Then SiO_2_ (1.29 g, 21.20 mmol) was added. The reaction mixture was stirred at 90 °C for 8 h. After filtration, the filtrate was evaporated under the reduced pressure. Purification of residue by column chromatography on silca gel (Hexane: EtOAc = 7: 1) gave compound **4** as a yellow solid (2.68 g, 86.1%). ^1^H NMR (DMSO-*d*_6_, 300 MHz) *δ*: 1.35 (s, 9H), 1.68 (s, 3H), 2.24 (s, 3H), 2.59 (d, *J* = 6.62 Hz, 2H), 3.66 (t, *J* = 5.28 Hz, 1H), 5.45 (s, 3H), 7.59 (s, 4H).

**(*S*)-*tert*-Butyl-2-(4-(4-chlorophenyl)-2,3,9-trimethyl-6*H*-thieno[3,2-*f*][1,2,4] triazolo[4,3-*a*][1,4]diazepin-6-yl) acetate ((+)-JQ1)**

To a solution of **4** (0.85g, 2.02 mmol) in THF (15 mL) was added potassium *tert*-butoxide (0.25g, 2.24 mmol) dropwise at -78 °C. The reaction mixture was warmed to -10 °C immediately and stirred for 30 min. Then, the reaction mixture was cooled to -78 °C and diphenyl chlorophosphate (0.35 mL, 2.44 mmol) was added. The stirred solution was allowed to warm to -10 °C and stirred for another 45 min. After addition of acetylhydrazide (0.23 g, 3.01 mmol), the reaction mixture was stirred at room temperature for 1 h. 1-Butanol (17.5 mL) was added and the solution was heated to 90 °C and kept for 1 h. After cooling to 20 °C, the solvent was evaporated. The residue was dissolved in CH_2_Cl_2_ (30 mL) and the organic phase was washed with saturated NaHCO_3_ solution (10 mL), brine (10 mL), dried over anhydrous Na_2_SO_4_ and concentrated under reduced pressure. Purification of residue by column chromatography on silca gel (Hexane: EtOAc = 8: 1) gave (+)-JQ1 as a white solid (0.56 g, 60.2%). ^1^H-NMR (DMSO-*d*_6_, 300 MHz) *δ*: 1.40 (s, 9H), 1.57 (s, 3H), 2.28 (s, 3H), 2.91 (dd, *J* = 16.50 Hz, 6.93 Hz, 1H), 3.09 (dd, *J* = 16.50 Hz, 6.93 Hz, 1H), 3.96 (t, *J* = 7.08 Hz, 1H), 7.42 (d, *J* = 8.34 Hz, 2H), 7.51 (d, *J* = 8.34 Hz, 2H), 11.19 (s, 1H).

**(*S*)-2-(4-(4-Chlorophenyl)-2,3,9-trimethyl-6*H*-thieno[3,2-*f*][1,2,4]triazolo[4,3-*a*][1,4]diazepin-6-yl) acetic acid (5)**

To a solution of (+)-JQ1 (0.50 g, 1.09 mmol) in CH_2_Cl_2_ (10 mL) was add trifluoroacetic acid (2.5 mL). The solution was stirred at room temperature for 2 h. The solvent was evaporated under the reduced pressure and toluene (5 mL × 3) was added to the residue. The solvent was evaporated under the reduced pressure and the process repeated three more times. The residue was dissolved in CH_2_Cl_2_ (5 mL × 2) and the solvent was evaporated under the reduced pressure. The process was repeated two more times. The resulting solid was further purified by recrystallization from EtOAc to give **5** as a white solid (0.41 g, 90.1%). ^1^H-NMR (DMSO-*d*_6_, 300 MHz) *δ*: 1.42 (s, 9H), 1.63 (s, 3H), 2.42 (s, 3H), 2.60 (s, 3H), 2.73 (s, 1H), 2.89 (s, 1H), 4.41 (t, *J* = 6.72 Hz, 1H), 7.42 (d, *J* = 8.58 Hz, 2H), 7.51 (d, *J* = 8.58 Hz, 2H).

**(S)-2-(4-(4-chlorophenyl)-2,3,9-trimethyl-6H-thieno[3,2-f][1,2,4]triazolo[4,3-a][1,4]diazepin-6-yl)-N,N-dimethylacetamide ((+)-ND)**

To a solution of **5** (0.40 g, 1.00 mmol) in DMF (5 mL) was added dimethyl amine (0.05 g, 1.02 mmol), HBTU (0.75 g, 2.00 mmol) and triethylamine (0.16 mL). The reaction mixture was stirred at room temperature for 2 h. After reaction, water (50 mL) was added to the mixture. The aqueous layer was extracted with EtOAc (10 mL × 3). The combined organic layer was washed with brine (10 mL), dried over anhydrous Na_2_SO_4_ and concentrated under reduced pressure. Purification of residue by column chromatography on silca gel (Hexane: EtOAc = 2: 1) afford (+)-ND as a white solid (0.24 g, 56.2%). ^1^H-NMR (DMSO-*d*_6_, 600 MHz) δ: 1.62 (s, 3H), 2.40 (s, 3H), 2.58 (s, 3H), 2.85 (s, 3H), 3.15 (s, 3H), 3.35 (q, 1H), 3.57 (q, 1H), 4.54 (t, *J* = 6.77 Hz, 1H), 7.42 (d, *J* = 8.68 Hz, 2H), 7.47 (d, *J* = 8.68 Hz, 2H).

Enantiomers (-)-JQ1 and (-)-ND were synthesized according to a similar procedure described in supplementary Fig. 1, by replacing the reagent Fmoc-L-Asp-OtBu with Fmoc-D-Asp-OtBu.

**The primers of qRT-PCR**

The primers used included: ATPase H^+^ Transporting V0 Subunit D2 (V-ATPase d_2_), forward 5’-AAGCCTTTGTTTGACGCTGT-3’ and reverse 5’-TTCGATGCCTCTGTGAGATG-3’; Dendrocyte expressed seven transmembrane protein (DC-STAMP) , forward 5’-GCTGTATCGGCTCATCTCCT-3’ and reverse 5’-AAGGCAGAATCATGGACGAC-3’; TRAP, forward 5’-CCATTGTTAGCCACTAACGG-3’ and reverse 5’-CACTCAGCACATAGCCCACA-3’; Atp6v0d2, forward 5’-AAGCCTTTGTTTGACGCTGT-3’ and reverse 5’-T TCGATGCCTCTGTGAGATG-3’; cathepsin K (Ctsk), forward 5’-CTTCCAATACGTGCAGCAGA-3’ and reverse5’-TCTTCAGGGCTTTCTCGTTC-3’; matrix metalloproteinase-9 (Mmp-9), forward 5’-CGTGTCTGGAGATTCGACTTGA-3’ and reverse 5’-TTGGAAACTCACACGCCAGA-3’; GAPDH, forward 5’-AGGTCGGTGTGAACGGATTTG-3’ and reverse 5’-GGGGTCGTTGATGGCAACA-3’.

**Reference**

1. Filippakopoulos P, Qi J, Picaud S, Shen Y, Smith W B, Fedorov O, et al. Selective inhibition of BET bromodomains. *Nature* **7327,** 468, 1067-1073 (2010).

**Spectral data**

**Copies of the NMR for representative compounds**

Compound **2** (In supplementary Fig. 1)


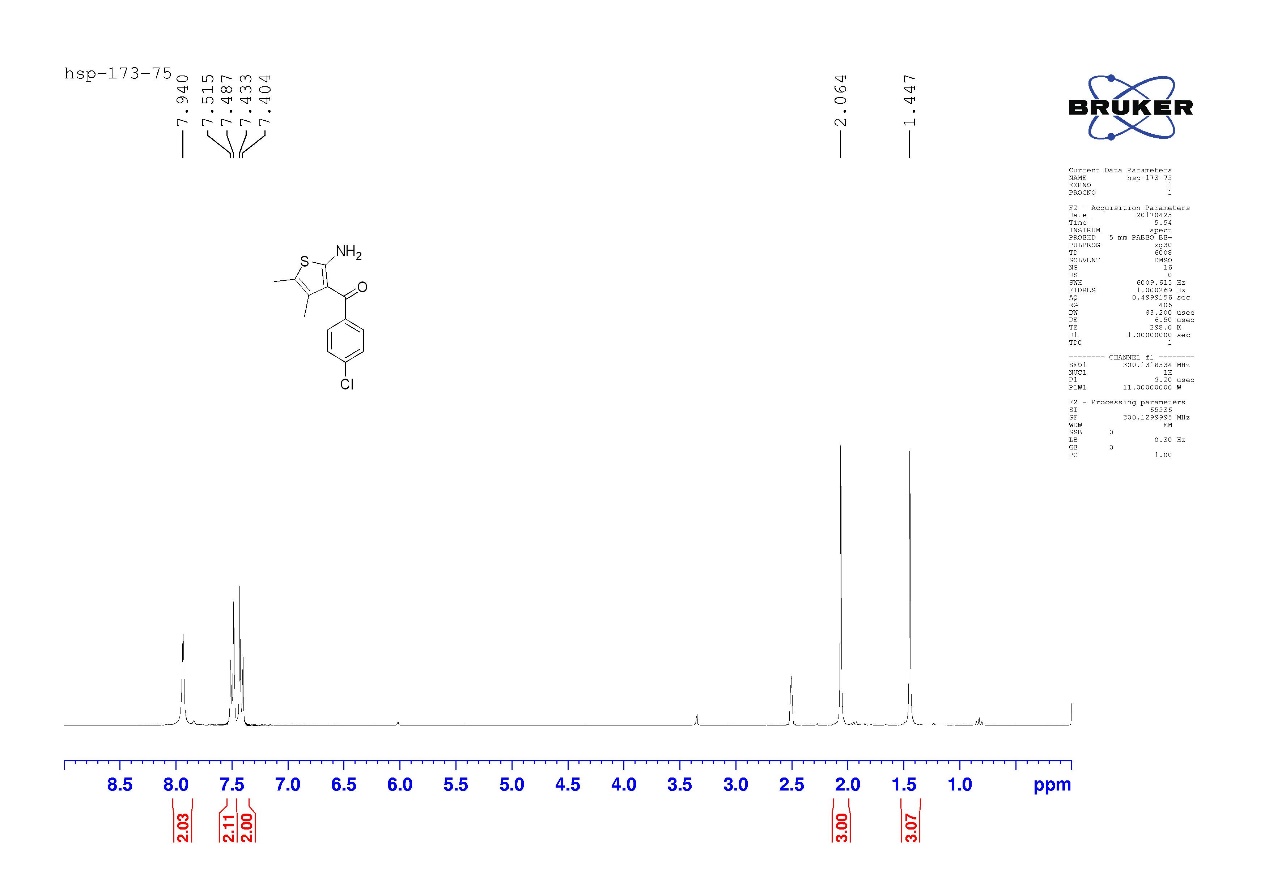


Compound **3** (In supplementary Fig. 1)


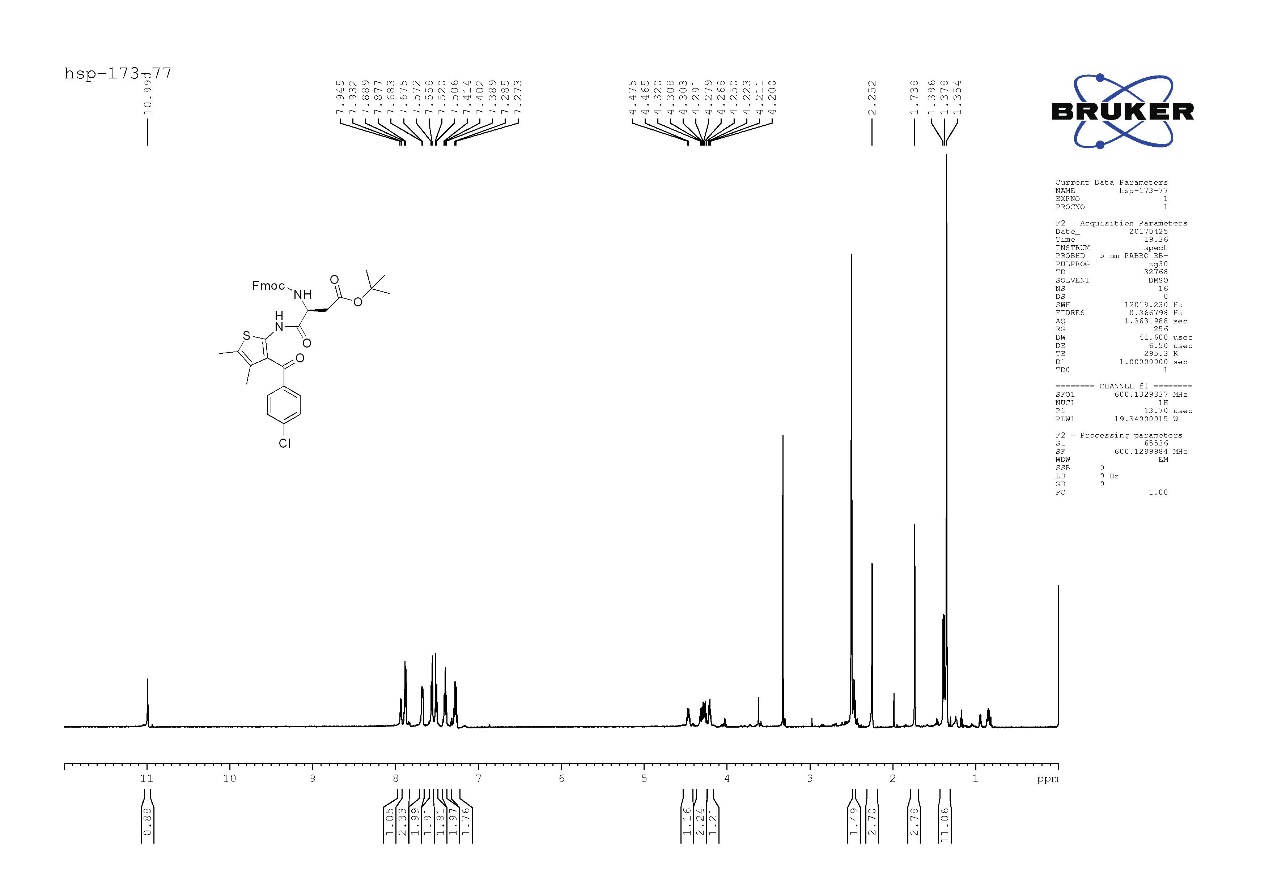


Compound **4** (In supplementary Fig. 1)


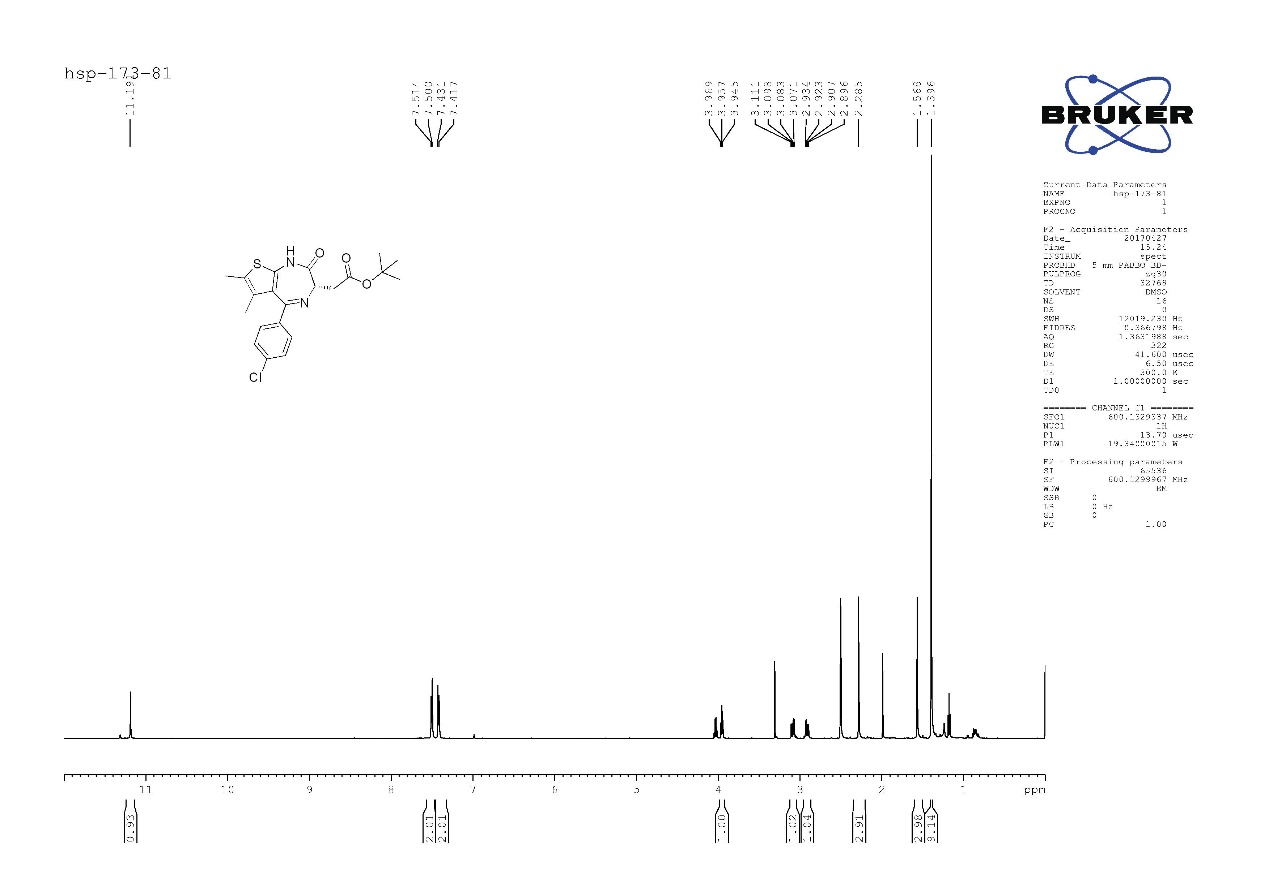


(+)-**JQ1** (In supplementary Fig. 1)


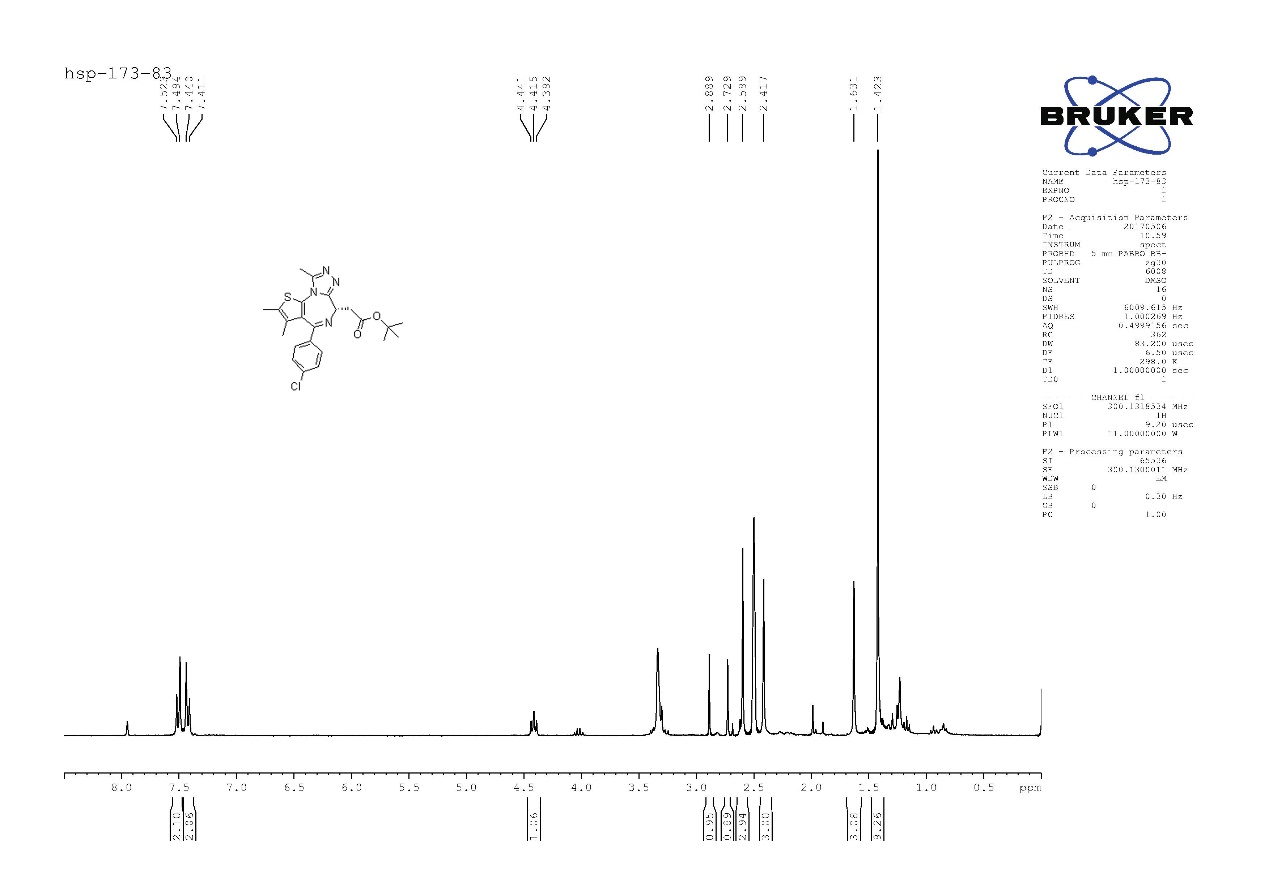


(+)-**ND** (In supplementary Fig. 1)


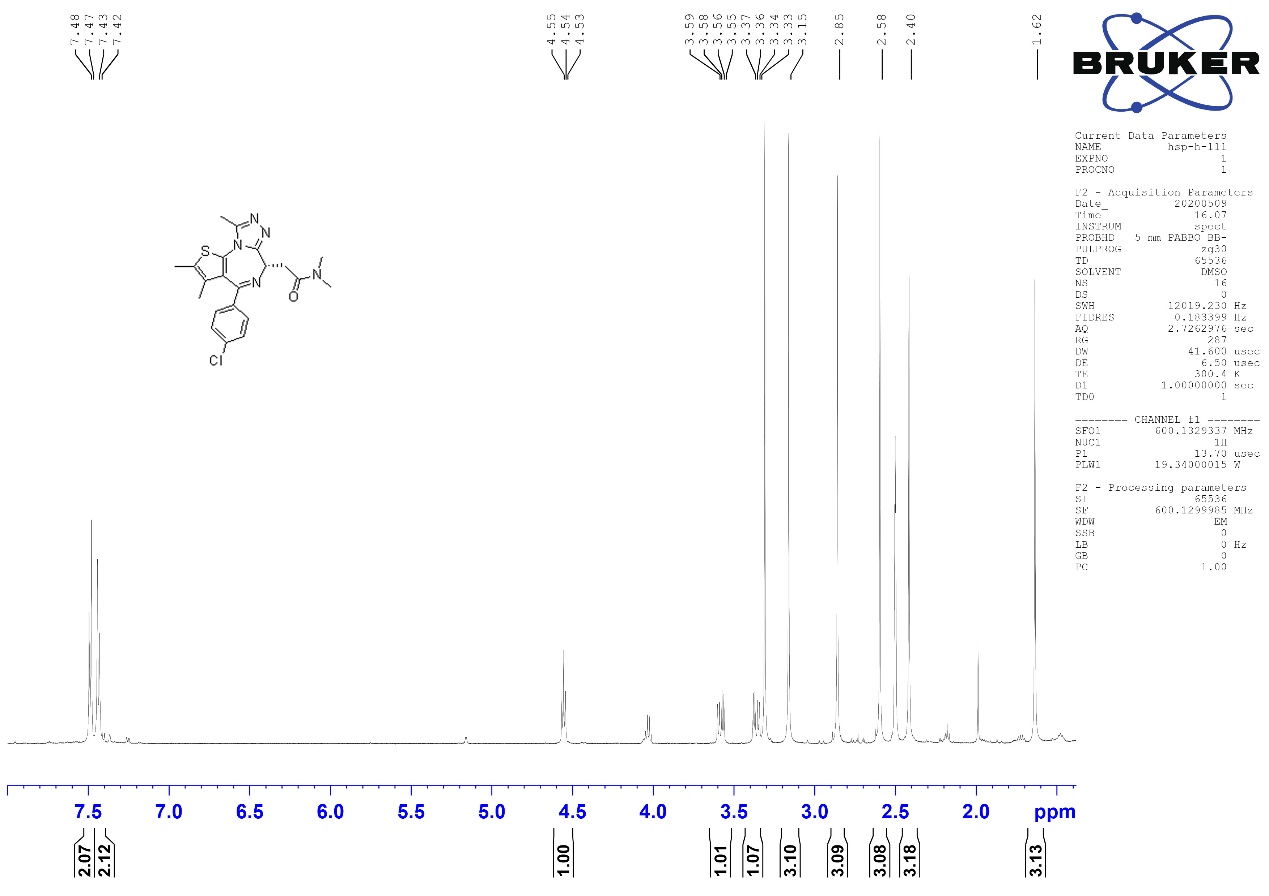

Supplement: Supplementary file 1 — Supplementary Materials and Methods [file 41419_2021_3939_MOESM1_ESM.docx]
