# Supplementary material for: A novel BRD4 inhibitor suppresses osteoclastogenesis and ovariectomized osteoporosis by blocking RANKL-mediated MAPK and NF-κB pathways
Source: Cell Death Dis. 2021 Jun 26;12(7):654. doi: 10.1038/s41419-021-03939-7 (PMC8236062; doi:10.1038/s41419-021-03939-7)
Supplement: Supplementary file 2 — Supplementary figure legends [file 41419_2021_3939_MOESM2_ESM.docx]

**Supplementary figure legends**

**Supplementary Fig. 1** Synthesis route for the (+)-JQ1 and (+)-ND.

**Supplementary Fig. 2** Surface diagram of the interactions between compounds with BRD4. **A** The binding mode of (+)-ND with BRD4 (PBD: 3MXF). **B** The binding mode of (+)-JQ1 with BRD4 (PBD: 3MXF).
