# Supplementary figures and images for: A novel BRD4 inhibitor suppresses osteoclastogenesis and ovariectomized osteoporosis by blocking RANKL-mediated MAPK and NF-κB pathways
Source: Cell Death Dis. 2021 Jun 26;12(7):654. doi: 10.1038/s41419-021-03939-7 (PMC8236062; doi:10.1038/s41419-021-03939-7)

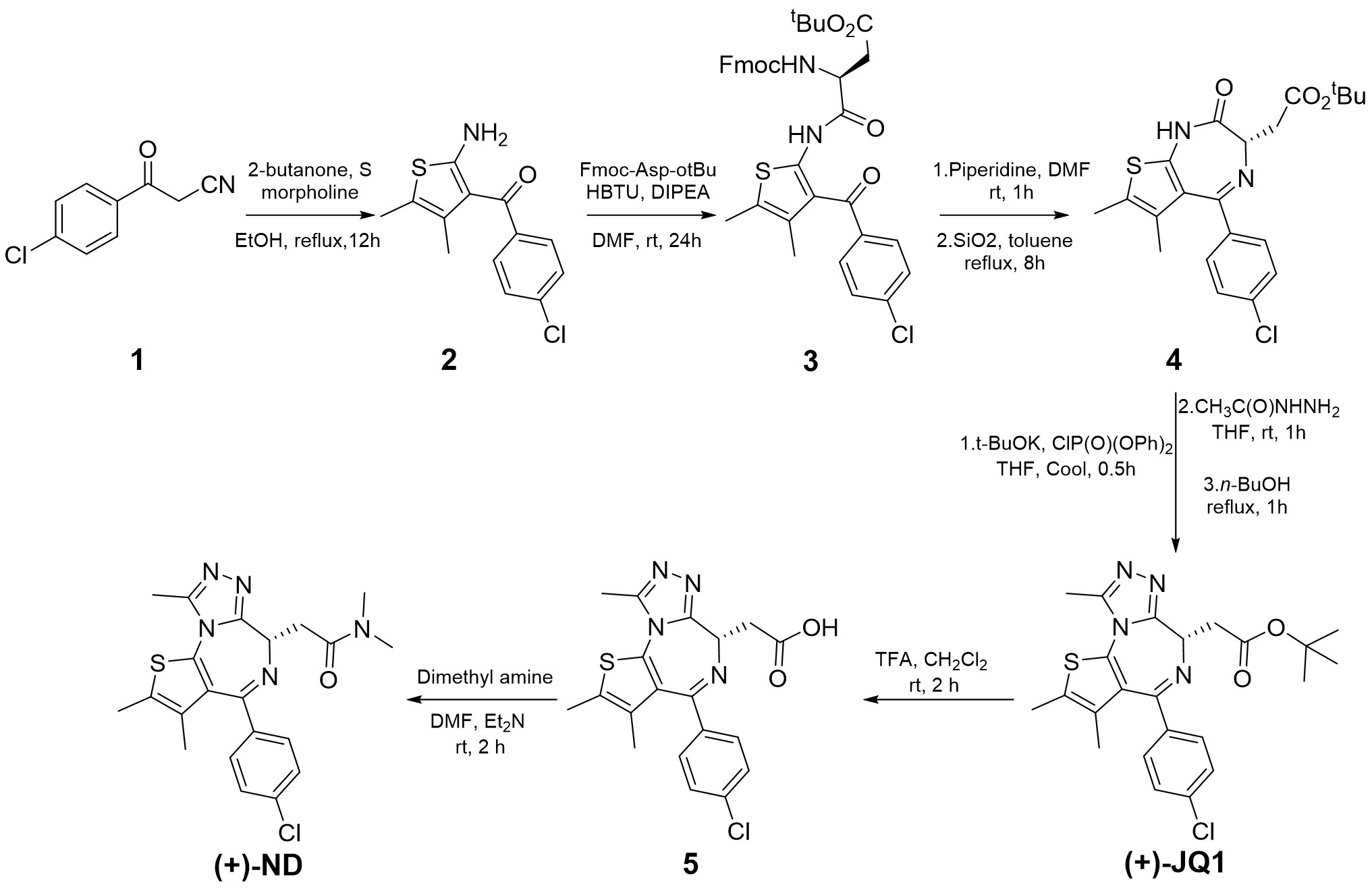

Supplement: Supplementary file 3 — Figure S1 [file 41419_2021_3939_MOESM3_ESM.tif]

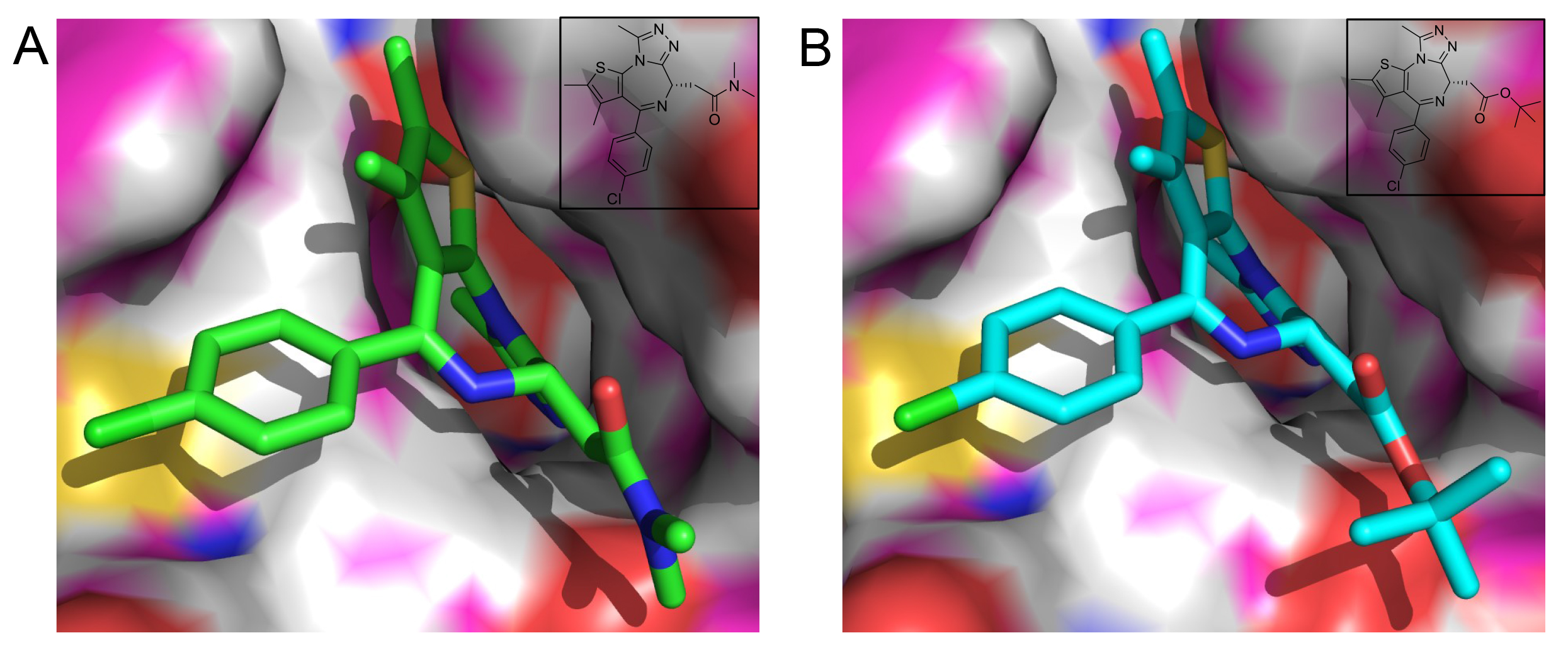

Supplement: Supplementary file 4 — Figure S2 [file 41419_2021_3939_MOESM4_ESM.tif]
